# Correlation and Influence of Seasonal Variation of Diet with Gut Microbiota Diversity and Metabolism Profile of Chipmunk

**DOI:** 10.3390/ani12192586

**Published:** 2022-09-27

**Authors:** Wei Teng, Iram Maqsood, Huan Wang, Jianzhang Ma, Ke Rong

**Affiliations:** 1 College of Wildlife and Protected Area, Northeast Forestry University, Harbin 150040, China; 2 Department of Zoology, Shaheed Benazir Bhutto Women University, Peshawar 25000, Pakistan; 3 Key Laboratory of Zoological Systematics and Evolution, Institute of Zoology, Chinese Academy of Sciences, Beijing 100101, China; 4 University of Chinese Academy of Sciences, Beijing 100049, China

**Keywords:** gut microbiota, metabolism, seasonal variations, diet composition, chipmunk

## Abstract

**Simple Summary:**

The Chipmunk (*Tamias Sibiricus*) plays a vital role in seed dispersal, but its seasonal diet, intestinal microflora structure, and metabolism profile are not clear at present, which is the main content of this study. Understanding the above contents is helpful in understanding further the factors affecting the chipmunk’s stability and population change. Our results showed significant differences in dietary habits, intestinal microflora structure, and nutrient absorption of chipmunks in different seasons. This study demonstrated that food in different seasons had essential effects on chipmunk’s intestinal microbe structure and metabolism profile.

**Abstract:**

*Tamias Sibiricus* is the only member of the genus Tamias, a significant and vigorous seed distributor and vital food for their predators. No information is known about the strict diet, gut microbiota structure, and metabolism profile of chipmunks and how they diversify seasonally. The above factors, as well as flexibility toward seasonal shifts, are critical in defining its growth rates, health, survivorship, and population stability. This study explored the diet, gut microbiota composition, and chipmunk metabolism. Additionally, the influence of different seasons was also investigated by using next-generation sequencing. Results revealed that seasons strongly affected a diet: streptophyte accounted for 37% in spring, which was lower than in summer (34.3%) and autumn (31.4%). Further, Ascomycota was observed at 43.8% in spring, which reduced to 36.6% in summer and the lowest (31.3%) in autumn. Whereas, nematodes showed maximum abundance from spring (15.8%) to summer (20.6%) and autumn (24.1%). These results signify the insectivorous nature of the chipmunk in summer and autumn. While herbivorous and fungivorous nature in spring. The DNA analysis revealed that chipmunk mainly feeds on fungi, including Aspergillus and Penicillium genus. Similar to diet composition, the microbiome also exhibited highly significant dissimilarity (*p* < 0.001, R = 0.235) between spring/autumn and spring/summer seasons. Proteobacteria (35.45%), Firmicutes (26.7%), and Bacteroidetes (23.59%) were shown to be the better discriminators as they contributed the most to causing differences between seasons. Moreover, PICRUSt showed that the assimilation of nutrients were also varied seasonally. The abundance of carbohydrates, lipids, nucleotides, xenobiotics, energy, terpenoids, and polyketides metabolism was higher in spring than in other seasons. Our study illustrates that seasonal reconstruction in the chipmunk diet has a significant role in shaping temporal variations in gut microbial community structure and metabolism profile.

## 1. Introduction

Researchers’ efforts are ongoing in acquiring animal diets as it is a prerequisite for understanding animal ecology, habitat requirements, ecosystem functioning, and finding out interactions between organisms as all are connected in one way or another [1,2,3]. Identifying each is a building block that builds larger webs and controls the flow of nutrients and energy through food webs [4]. The most significant challenges are for those predators that feed on many different species, such as herbivores, generalists, and omnivores, that can harvest different species of plants and animals. Besides this, many predators show opportunistic and plastic feeding behaviours that may vary with time and space, depending on the availability of the resource, requirement of nutrition and, condition of a particular environment and that may exhibit an intraspecific variation [5,6,7].

In this study, we focus on Tamias Sibiricus, the only member of the genus Tamias. Naturally, it is native to northern Asia and distributed from central Russia to China, which is also found in Korea and northern Japan. Eastern Europe is also the home for Tamias Sibiricus because of the individuals escaping from captivity [8].

Siberian chipmunks are the source of vital food for their predators. Chipmunks’ behaviour in buried and forgotten caches makes them vigorous seed distributors. It is recommended that chipmunks also assist in the dispersal of fungal spores. Despite the knowledge of their particular keystone function in harvesting and seedling establishment [9], the information about a chipmunk’s diet is fragmented due to the challenge of observing and identifying the species that the chipmunk digests. Additionally, no data is known about how the chipmunk diet diversifies seasonally. Thus, we investigated the diet behaviour of Siberian chipmunks for periods of three seasons (e.g., spring, summer, and autumn) in their native range. Previously reported, these small mammals justify several vital functions in forest ecosystems as they ingest many diverse fungi, including those involved in symbiotic mycorrhizal associations with trees and responsible for the distribution of subterranean sporocarps (truffles), which have lost the competence to spread their spores through the air [10].

Besides our poor knowledge about the seasonal patterns of the chipmunk diet, we are also unaware if the chipmunk gut microbial communities fluctuate in reaction to changes in diet. A few studies have found an apparent association between the seasonal patterns of changes in gut microbiota composition in herbivores with fluctuations in food [11,12]. Suppose these temporal Shifts in gut microbial communities are connected with animal dietary shifts. In that case, this will propose that nutritional quality, the diet’s species composition, and anti-nutritional secondary metabolites cause changes in gut microbes’ community to facilitate food processing [13,14]. Additionally, it has been shown that the gut microbiome shows mutualistic relations with the host by affecting the host’s digestion and ability to assimilate nutrition. This phenomenon accommodates the host with energy and nutrients [15]. For example, to meet physiological needs, gut microbes can assist a host to break down indigestible constituents such as hemicellulose and cellulose, transforming them into useable compounds [16]. Variations in gut microbiome structure are generally reflected in microbial reactions to selective pressures enforced by changes in the host’s diet, physiology, and health [17,18].

In the current study, we used DNA analyses from chipmunk faeces contents, which can illustrate trophic interactions at advanced resolution than any other method, thus producing new techniques to quantify diets and gut microbiomes in a better way. After the emergence of high-throughput sequencing (HTS) technologies, the meta-barcoding approach was adopted in which every prey DNA (barcodes) in a sample could be sequenced. It has the potential to provide diet information to a maximum resolution at the the species or genus level and even allow possible differentiation among strains [19]. In past years, massive development in both the methodology and application of microbiome and dietary tracing methods has been explored. Previously, various diet tracing methods such as visual analyses of stomach, gut or scat content [20], microscopic examination of faeces, fatty acid and alternative biomarkers, cheek contents, or alimentary canal, stable bulk isotopes [21], plant alkane fingerprints, compound-specific stable isotopes [22] and stable isotope analysis, protein electrophoresis of gut contents, and reflectance spectroscopy [23,24,25,26] have been adopted. Most of them are difficult or impossible to carry out when dealing with animals fed in complex environments with many species [3,19,27]. Even more, previously unknown trophic relationships can be resolved by metabarcoding, even assisting in excavating information about organisms that were traditionally impossible or difficult to recognize using visual methods [19,28]. This molecular method has emerged as a popular option for characterizing trophic interactions because of its ability to provide high taxonomic resolution of prey and their sensitivity to rare, highly degraded items and those that leave no visual trace, such as liquid feeding [29]. It is a cost and time-efficient tool to acquire detailed animal diet and microbiota information [3,19]. 

We believe our study will enlighten future management plans and conservation actions for prey or predator and assist in understanding ecosystem functioning as rodents play an essential role in ecological ecosystems broadening from tropical forests to arctic tundra [30,31,32,33,34]. Further, rodents also influence the structure of terrestrial populations by using seeds, vegetation, and animals as prey. For example, in the American Southwest, the granivore rate of kangaroo rats (*Dipodomys* spp.) determines plant communities, whether grasses or shrubs dominate them Other studies have found that rodents have a significant impact on primary production, the water cycle, and animal community dynamics [35,36,37]. Studies proved that rodents found in tropical and temperate forests could cause a switch in tree recruitment patterns by their actions as seed dispersers or seed predators [38,39,40]. This fact-finding will enrich the biological background knowledge of Siberian chipmunks’ diet, gut microbial composition, and metabolism profile and elucidate their seasonal fluctuation. Our study has figured out that seasonal deviations in diet trigger a shift in gut microbial composition and host metabolism. Subsequently, it concluded that high-throughput sequencing had enabled significant enhancement in understanding omnivorous diets, gut microbiome structure, and host metabolism. Our research will potentially assist researchers in exploring which season is vital for which food item and which season is essential for specific plant seed dispersal.

## 2. Material and Methods

### 2.1. Field Sampling

**Figure 1:**
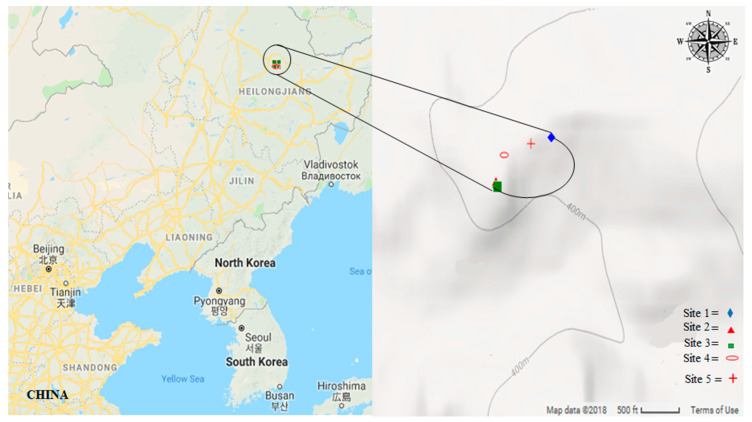
The regions and sampling sites of this study area. The left map shows the location of the Heilongjiang province. On the right map, the grey-coloured map is the sampling site for chipmunk diet analysis.

The chipmunk was trapped between April and October 2017 in the Liangshui National Nature Reserve in the Mountains of Heilongjiang Province of northeastern China (Figure 1). Five study areas were sampled (Table 1). The study area was dominated by Korean pine trees, containing mixed broadleaf-conifer forests. Pine parent forest was also present in remnant patches, which reported approximately 2375 ha distributed on the edges. 

All traps were baited with cotton dipped in vegetable oil. Samples were collected randomly from individuals who may or may not have been the same individuals from one trip to the next. Fresh faecal samples were collected on parchment paper inside the cage, transferred to EP tubes, and stored at −20 °C until DNA extraction. The samples were then transported to Shanghai Personal Biotechnology Co., Ltd (Shanghai, China). for diet, microbiome, and metabolism analysis. Faecal samples were collected twice per month for three consecutive days and divided into three seasons: spring, summer, and autumn.

### 2.2. Moral Statement 

This sample collection procedure has been reviewed and granted permission by Liangshui National Nature Reserve Station. This investigation did not contain endangered or protected species. Animals were handled following the guidelines established by the American Society of Mammologists [41]. The animal ethics committee of the college of wildlife and protected area, Northeast Forestry University, approved under the approval number of WPAAC107 dated 17 March 2017.

### 2.3. DNA Extraction

Non-invasive faecal samples were handled carefully in the laboratory to avoid contamination from other DNA samples. To extract DNA from faeces, sterile swabs were dipped in thawed faecal samples before being placed in reaction wells. DNA was retrieved using the MoBio PowerSoil^®^ DNA Isolation Kit(12888) (Invitrogen, Waltham, MA, USA, (75510-019) [42]. Extracted DNA run on 0.8% agarose gel electrophoresis for confirmation. DNA quantitation was performed by the Quant-iT PicoGreen dsDNA Assay Kit (Invitrogen, Waltham, MA, USA) and verified the DNA quality and quantity for subsequent PCR experimentation. Blank extractions without samples were systematically performed to monitor possible contamination. 

### 2.4. DNA Amplification and Sequencing

We amplified the rbcL coding sequences from plastids as the “core” DNA barcode (Table 2) [43] to infer which plant species the chipmunk had consumed, the gene encoding mitochondrial cytochrome c oxidase I (COI) for animal-based diet consumption [44] and the ITS region for fungi exposure. The V4 region of the 16S rRNA gene was amplified by the primer set, which is well suited to the accurate phylogenetic placement of bacterial and archaeal sequences [45]. Together, these primers are expected to amplify nearly all bacterial and archaeal taxa with few biases [46]. Furthermore, we investigated the fluctuations in the relative abundance of diet items over time and explored their effect on the gut microbiome and host metabolism profile.

All DNA amplifications were carried out in a final volume of 25 µL, using 2 µL of DNA extract as a template. The amplification mixture contained 0.25 of Q5 high-fidelity DNA polymerase, 5 µL 5* Reaction Buffer, 5 µL 5* High GC Buffer, 2 µL dNTP (2.5 mM), 1 µL of each primer (10 uM), and 8.75 µL of ddH2O. The PCR mixture was initially denaturated at 98 ℃ for 2 min. We set the system for 25–30 cycles, denaturation at 98 ℃ for 15 s, annealing at 55 ℃ for 15 s, extension at 72 ℃ for the 30 s, final extension at 72 ℃ for 5 min, and finally hold at 10 ℃. The primer pair containing a 7-bp barcode unique to each sample was used. The PCR amplification product was detected by 2% agarose gel electrophoresis, and the target fragment was purified using a gel recovery kit (AXYGEN, Sigma Aldrich, St. Louis, MO, USA).

A sequencing library was prepared using Illumina’s TruSeq Nano DNA LT Library Prep Kit (Illumina, Hayward, CA, USA). The DNA fragmentation was performed by excising at the 5′ end of the DNA sequence and repairing it using End Repair Mix 2 in the kit. At the same time, a phosphate group was added to fill in the missing base at the 3′ end. Then follow the addition of the A base to the 3′ end of the DNA fragment to ensure that the target sequence ligates to the T base at the 3′ end of the sequencing adapter. A library-specific sequencing adapter to the 5′ end of the DNA fragment was added to allow the DNA molecule to be immobilized on the flow cell. Further, the sequencing library was enriched by additional PCR amplification. Library-enriched products were again purified using BECKMAN AMPure XP Beads (Agencourt Bioscience, La Jolla, CA, USA). Final library selection and purification were conducted by 2% agarose gel electrophoresis. After quantification and pooling, the amplicons were sequenced on an Illumina MiSeq instrument at the Shanghai Personal Bio Company (Shanghai, China) with the 2 × 300 bp paired-end protocol [47]. Sequence data were processed using the QIIME (Quantitative Insights Into Microbial Ecology, v1.8.0, http://qiime.org/) pipeline (accessed on 20 April 2017) [48]. Sequences were assigned to specific samples based on their unique barcodes, and sequences were clustered at 100% similarity for each taxon. These primer concentrations have been chosen after a series of test experiments with various concentrations of PrioB (data not shown). 

### 2.5. Sequence Analysis and Taxon Assignation

The query sequence was first identified using the QIIME software (Quantitative Insights Into Microbial Ecology, v1.8.0, http://qiime.org/, accessed on 20 April 2017) [48]. Sequences shorter or equal to 150 bp in length and containing any ambiguous bases were excluded. We also trimmed: (1) reads with more than one bp mismatch with a 5′ primer; (2) the reads contain homopolmers longer than eight bases. USEARCH (v5.2.236, http://www.drive5.com/usearch/, accessed on 20 April 2017) was invoked via the QIIME software (v1.8.0, http://qiime.org/, accessed on 21 April 2017) to check and delete the chimera sequences. Through QIIME software, the UCLUST sequence alignment tool [53] was adopted to merge and define the OTUs from the previously obtained sequences based on 97% sequence similarity, and the sequence with the highest abundance in each OTU was selected as the representative sequence of the OTU. 

Further, global singletons (i.e., OTUs represented by only a single sequence over an entire dataset) and OTUs abundance with less than 0.001% (one hundred thousandths) of total sample sequences were removed for a subsequent series of error-free analyses [54,55]. To assign a taxon to each of the filtered sequences, we successively used different taxonomic reference libraries. We performed alignment searches against 16S rRNA gene sequences present in the Green Gene database (Release 13.8, http://greengenes.secondgenome.com/, accessed on 25 April 2017) [56], the RDP (Ribosomal Database Project) database (Release 11.1, http://rdp.cme.msu.edu/, accessed on 25 April 2017) [57] and the Silva database (Release 115, http://www.arb-silva.de, accessed on 25 April 2017) [58] for bacteria and archaea. We used the UNITE database (Release 5.0, https://unite.ut.ee/, accessed on 25 April 2017) [59] of ITS sequences for fungi. We performed Basic Alignment Search Tool BLAST-searches against sequences in the NCBI GenBank (http://www.ncbi.nlm.nih.gov/, accessed on 25 April 2017) for the rbcL and COI genes. Resultant identification at the genus level was only accepted if the sequence similarity with the best match exceeded 97%.

### 2.6. Metabolic Activity of the Bacterial Communities

A bioinformatics tool, PICRUSt (Phylogenetic Investigation of Communities by Reconstruction of Unobserved States) Galaxy version (1.0.0), created by Lingille et al., in 2013, USA [60] was used to explore the functional composition of a microbial community found in the gut contents of a chipmunk concerning different seasons. The functions were categorized at level 2 and were generated by the KEGG (Kyoto Encyclopedia of Genes and Genomes) pathway.

### 2.7. Statistical Analysis

Data normality was calculated using the Shapiro-Wilk test, Anderson-Darling A test, and Jarque-Bera JB. After the normality test, no significant departure from normality was found. The Shannon diversity index [61] and the Chao1 richness estimator [62] were also calculated. The participation of each taxon type in the average dissimilarity among the seasons was computed by operating the similarity percentages procedure (SIMPER) [63]. Analysis of similarity (ANOSIM), on a Bray–Curtis similarity data was computed on the observed diet and gut microbiome data using 9999 permutations to investigate the statistically significant dissimilarity in diet composition and gut microbiome structure among samples acquired during spring (April and May), summer (June, July, and August) and autumn (September, October) seasons. PCA was used to observe dissimilarity using Euclidean distance and visualize correlation among seasons at the genus level. Analysis of similarities (ANOSIM), SIMPER [63], data normality, and PCA were calculated using PAST (PAlaeontological STatistics) ver. 3.17 (USA) [64].

## 3. Results

### 3.1. Diet

**Figure 2:**
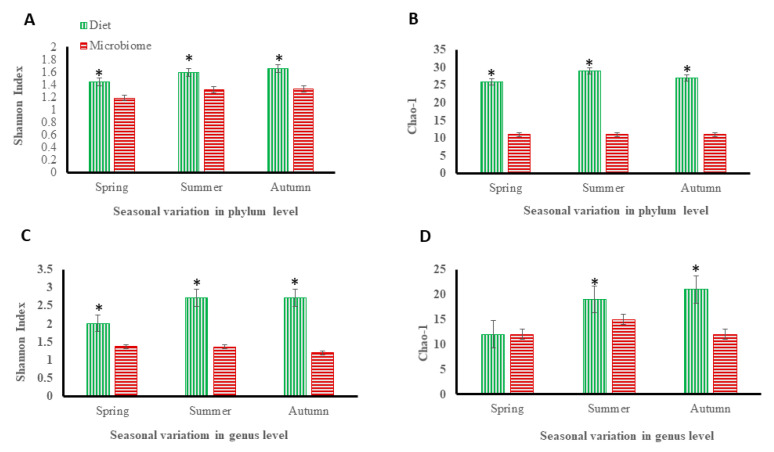
Comparison of seasonal variation in Chipmunk Diet and the gut microbiome. Chao1 richness estimator (Chao1) and Shannon biodiversity index were calculated for each season at the phylum and genus levels. (**A**) = Chao-1 for diet at phylum and genus level; (**B**) = Shannon Index for diet at phylum and genus level; (**C**) = Chao-1 for microbiome at phylum and genus level; (**D**) = Shannon Index for microbiome at phylum and genus level. * indicate significant difference (*p* < 0.05).

Deviations in the relative abundance of DNA sequences were used to conclude differences in prey ingestion among different seasons. Total dissimilarities in prey consumption were inferred by comparing matrices of pairwise proportions among prey taxons present in each population. Across three seasons, analysis of chipmunk’s faeces revealed 27 phyla representing up to 33 different genera in the chipmunk’s diet. Dietary composition varied seasonally (*p* < 0.001, Figure 2A, Supplementary Files S1 and S4). Ascomycota (43.8% of sequences on average), Streptophyta (37.0%), Nematoda (15.8%), Basidiomycota (2.1%), Arthropoda (0.3%), Glomeromycota (0.2%) and Zygomycota (0.1%) are the dominant phyla that were consumed by chipmunk across three seasons. In the spring, the most abundant phylum of Ascomycota, which included the genera Aspergillus, Penicillium, and Aureobasidium, and the phylum Streptophyta, which included the genera Enemion and Hylomecon, were observed. The maximum abundance of phylum Basidiomycota, including genus Hymenogaster and phylum Nematoda, consisting of genus Travassostrongylus, Oswaldocruzia, Murshidia, and Steinernema, was observed in the autumn diet (Files S1).

We employed the Chao1 estimator of total richness to estimate the number of phyl and genus present in the samples (Figure 2A,C). The Shannon index (H) that correlates positively with species richness and evenness was also calculated at both phylum and genus levels (Figure 2B,D). Overall, the Chao1 and Shannon diversity indices indicated the great richness in diet items. We observed maximum α-diversity in the autumn season (Figure 2); in contrast, the spring season had a significantly higher variance in its α-diversity compared to the summer and autumn seasons. The diet during the summer and autumn seasons were similar, but variation was observed in the spring. In contrast to the analysis at the phylum and genus levels, the diet richness and biodiversity were maximum in autumn.

A SIMPER and ANOSIM test recognized significant differences, indicating an overall variation in relative proportions of prey items. Similarity percentages (SIMPER) exploration reveals the amount of input of each taxon based on dissimilarity observed between groups. It allowed us to identify which phylum and genus were most significant in generating the observed pattern of dissimilarity. All samples were pooled to perform one overall multi-group SIMPER and consumed the Bray-Curtis measure of dissimilarity, relating the individual sample in spring to the individual sample in summer and autumn. SIMPER also let us identify the taxon that was likely to be the dominant contributor to any difference between assessed seasons (Supplementary File S3). At the phylum level, the overall observed average dissimilarity in diet among seasons was 21.87% and the overall average Bray-Curtis dissimilarity at the genus level among seasons was 69.94%. At the phylum level, the Nematoda (24.09%), Ascomycota (17.43), Basidiomycota (11.02%) and Apicomplexa (3.27%) contributed most to the differences between season’s diets (Supplementary File S3). ANOSIM showed more dissimilarity at the genus level compared to phylum level. Diet composition at phylum level showed a significant difference between summer and autumn seasons (SIMPER, Euclidian distance, *p* < 0.05, *p* = 0.04, R = 0.1949, Permutation N: 9999) but illustrated highly significant dissimilarity between autumn and spring season (SIMPER, Euclidian distance, *p* = 0.001, Permutation N: 9999). Genus level diet composition is highly significant (*p* < 0.001, R = 0.2354) between spring/summer and spring/autumn.

**Figure 3:**
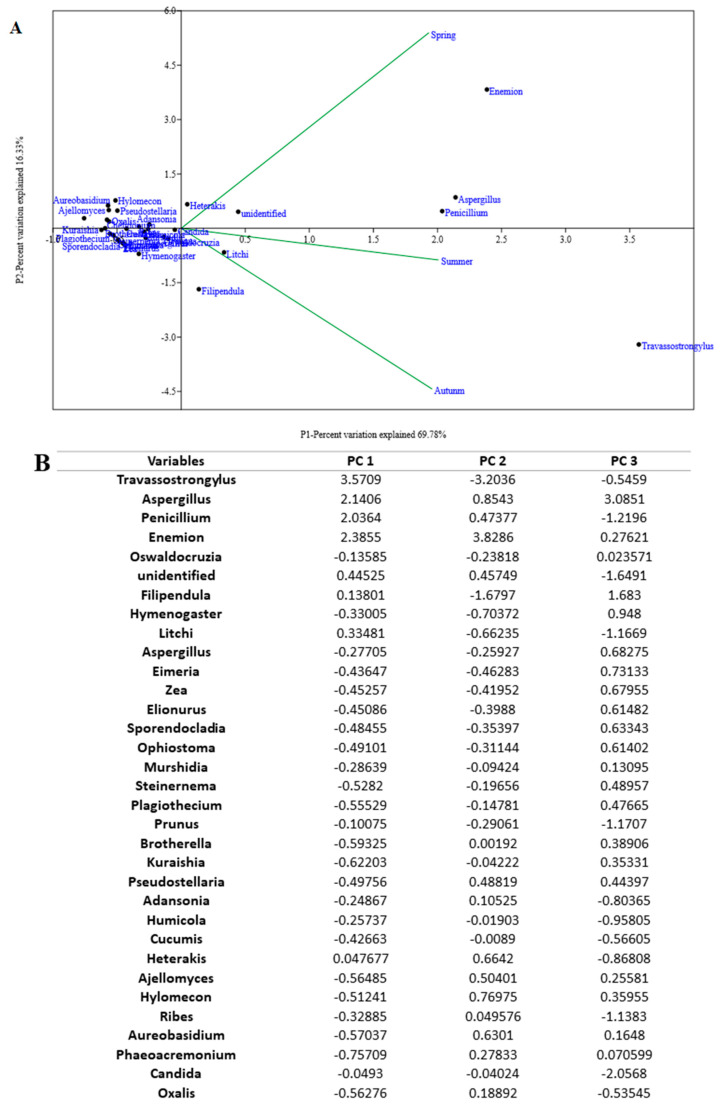
Diet variation among Seasons was detected by Principal component analysis (PCA). (**A**) Correlations of diet relative to the season were observed at the genus level. Dot represents the prey consumed by chipmunk and lines represents a seasonal pattern of diet changes seen in the chipmunk diet. PC1 explained 69.78% of the variation and PC2 explained 16.33%. (**B**) Component loading derived from PCA analysis of diet at the genus level.

Principal component analysis (PCA) was used to emphasize variation and bring out strong patterns of correlation among seasons. Bi-plot was used to assess the data structure. The results revealed that spring is more dissimilar from the other two seasons, and summer is more similar to autumn. Among diet items, Travassostrongylus, Aspergillus, Penicillium, and Enemion are the key genus cause dissimilarity among seasons, PC1 shows 69.78% variance, whereas PC2 shows 16.33% variance (Figure 3A).

### 3.2. Microbiota

**Figure 4:**
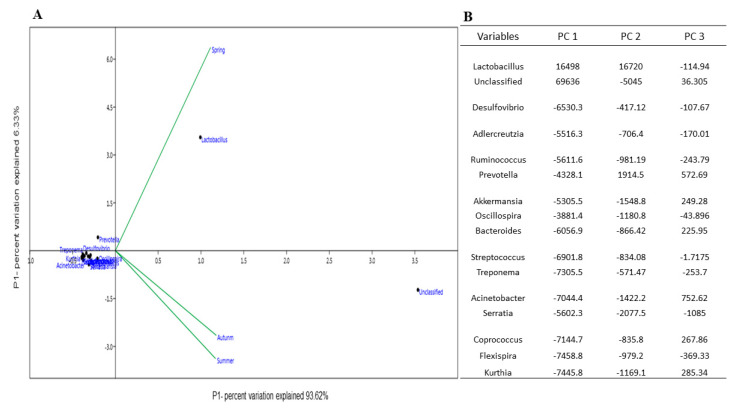
(**A**) Principal component analysis (PCA) revealed Gut microbiome variation and correlation among Seasons. This correlation was observed at the genus level. Dots and lines represented the microbiome community in the gut of chipmunks represent a seasonal pattern of microbiome variation seen in a chipmunk. PC 1 explained 93.62% variation and PC2 represented 6.33% variation in the gut microbiome community among the three seasons. (**B**) Components of gut microbiome at genus level were derived from PCA analysis.

Chipmunk’s gut microbiome unveiled 10 phyla, including up to 15 different genera. Gut microbiome composition varies seasonally, and a summary of all the comparisons made in this way is given in Figure 2 and Figure 4, Supplementary File S2 (*p* < 0.001). The four most abundant microbial phyla were Firmicutes (55.2%), Bacteroidetes (30.4%) and Proteobacteria (6.8%). Firmicutes consisting of Lactobacillus genus, Bacteroidetes comprised of Prevotella genus, and Proteobacteria containing Desulfovibrio genus were the more abundant phyla in the spring. These phyla exhibited a significant temporal change in relative abundance over the three seasons, decreasing more than two-fold from the gut microbial community in spring. We observed that the a-diversity of the gut microbiome of chipmunks varied seasonally (*p* < 0.001, Supplementary Files S2 and S3). 

A preliminary Principle Component Analysis (PCA) was conducted to visualize differences in bacterial genus composition between seasons and to determine which genus was most strongly associated with the differences observed. PCA confirmed that samples from the spring formed a distinct position in the ordination plot, and summer was similar to autumn. This separation was most apparent along the PC1 axis, which explained 93.62% of the overall variation, and for which Lactobacillus had maximum dissimilarity among seasons and was abundant in spring (Figure 4). The PC2 axis explained only 6.33% of the overall variation; however, no distinctions between seasonal groups were made through this component.

Differences in ß-diversity (i.e., SIMPER and ANOSIM) were also measured. When all time points were averaged together, there was significant variation among the three seasons. SIMPER analyses across phylum and genus levels were employed to identify taxa with the highest contribution to differences between the diet types. Further, SIMPER analyses across phylum and genus levels were employed to identify taxa with the highest contribution to differences between the seasons. (Supplementary File S3). The distinction between diet types was more apparent as the taxonomic level became more specific where SIMPER detected 34.37% dissimilarity at the phylum level, which increased to 35.22% dissimilarity at the genus level. At the phylum level, Proteobacteria (35.45%), Firmicutes (26.7%) and Bacteroidetes (23.59%) were shown to be better discriminators as they contributed most to differences between seasons, which indicates that the gut microbiome undergoes dramatic seasonal fluctuations. When we compared three seasons, significant (SIMPER, Euclidian distance, *p* < 0.05, *p* = 0.015, R = 0.0730, Permutation N: 9999) dissimilarity was observed between the autumn and spring seasons at the phylum level. Similar to diet composition, the microbiome showed highly significant dissimilarity (*p* < 0.001, R = 0.235) between spring/autumn and spring/summer seasons at the genus level. The gut microbiome showed no statistical difference (SIMPER, Euclidian distance, *p* > 0.05, *p* = 0.59, Permutation N: 9999) between the summer and autumn seasons.

### 3.3. Predicted Gut Microflora Function Using PICRUSt

**Figure 5:**
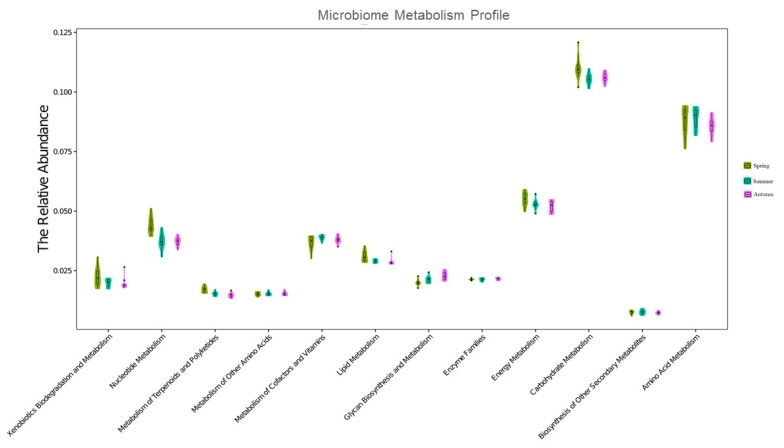
Comparison in the relative abundance of PICRUSt-generated functional profile of gut microbiota among three seasons.

It is unclear whether seasonal variation in the gut microbiome affects host metabolism. To understand the specific effects of the gut microbiome on host metabolism, PICRUSt was performed to predict the chipmunk gut microbiome functions, which showed that chipmunks in different seasons exhibited some differences in metabolism abundance at level 2, including carbohydrate, protein, amino acid, Xenobiotics, energy, cofactors and vitamins, glycan, lipid, terpenoids, and polyketides metabolism. A Violin Plot was used to visualize the distribution of the data and its probability density (Figure 5). The abundance of carbohydrates, lipids, nucleotides, xenobiotics, energy, terpenoids, and Polyketides metabolism was higher in spring than in summer and autumn.

The difference between spring and autumn is more significant (ANOSIM, *p* = 0.0005) as compared to spring and summer (ANOSIM, *p* < 0.001, *p* = 0.005, Euclidian R = 0.20). Further, an insignificant Euclidian difference (*p* = 0.29) was also investigated while comparing summer with autumn. In addition, Bray-Curtis dissimilarity (3.627%) among three seasons was illustrated through SIMPER analyses as shown in Supplementary File S4. These analyses supported us in figuring out the relationship between seasonal metabolism and microbiome profile.

## 4. Discussion

In this study, we assessed the first precise dietary habit of a small, striped rodent called the Siberian chipmunk, which belongs to the family Sciuridae [8]. With the help of the extensive local barcode library, we were able to accurately and reliably identify 33 highly abundant dietary genera from the faecal samples. No information was known about the seasonal variations of the diet, gut microbiota, and metabolism of the Siberian chipmunk. Therefore, we explore the seasonal shift in diet that leads to the alteration in gut microbiome diversity and host metabolism profile. Based on the preliminary results obtained in this study, we can conclude that Siberian chipmunk feed mainly on Ascomycota (fungivorous), Streptophyta (herbivorous) and Nematoda (insectivorous), supports the expectation that such a small mammal requires highly nutritious foods. This diet pattern shown by chipmunks is not consistent with the view that the Siberian chipmunk feeds mainly on plant material [65,66]. When we compared them, seasons showed greater flexibility in their diet patterns. Although they overlap in diet among the three seasons, in spring, Ascomycota (fungi), Streptophyta (plant) and Nematoda (insect) are the dominant phyla (presence in 43.8%, 37.0%, 15.8%, respectively). Within these phylum, genus Aspergillus (20.7%), Penicillium (13.6%), Enemion (28.7%) Travassostrongylus (9.9%) dominate, respectively. While in summer and autumn, Siberian chipmunks supplement their diet with an increasing proportion of Nematoda from 20.6% to 24.1%, which comprises the genus Travassostrongylus, Murshidia, and Steinernema, which is consistent with the previous studies [66,67].

Further, in response to seasonal variation in the chipmunk diet, we also observed seasonal variation in gut microbiota and host metabolism. Previously, High-throughput sequencing of the 16S rRNA gene from faecal samples has scrutinized gut microbiota at the phylum level. In this study, we also adopt HTS to describe the gut microbiota composition and explore the effect of seasonal variation in gut microbiota down to the genus level. The chipmunk gut microbiota at the phylum level is similar to other mammals (including humans) with two major groups, the Firmicutes and Bacteroidetes, that account for ~90% of the 16 S rRNA gene sequencing reads [68,69]. We found high levels of the Lactobacillus genus belong to the phylum Firmicutes, which is analogous to other omnivorous mammals, such as a wild mouse, bears, and lemurs. As in other mammals, Lactobacillus constitutes up to one-third of the microbiome community [70]. These results advocate that the gut microbiota of mammals can assemble regardless of the host species [68,69], reflecting a specific set of microorganisms that have fitted to life in the gastrointestinal tract [71]. We also observed in our study that diet is directly involved in shaping the gut flora as a maximum abundance of Firmicutes phylum in spring was observed, which was abundant with the genus Lactobacillus. Firmicutes in the gut of the obese were positively correlated with the increased capacity to harvest energy from the diet, which let them harvest more energy and more efficiently absorb calories that ultimately helped in weight gain [72]. This shows that diet influences the shifting of bacteria that have a proper role in the metabolism of the available dietary contents. Furthermore, a higher proportion of Firmicutes to Bacteroidetes has established a correlation with diet-induced or genetically obese mice [73,74,75].

The findings of this study illustrated that Lactobacillus was detectable in the gut microbiome in any season (Figure 4B). The amount of Lactobacillus correlates directly with the body fat ratio used to consume a high-sugar/high-fat diet [76]. We found that Lactobacillus directly correlates with fat and carbohydrate metabolism in chipmunks in three seasons. The minimum abundance of Lactobacillus directly accompanied protection from metabolic disorders in the obese. The possible mechanism behind this protection was the alteration of bile acid concentration in the lumen [77]. 

Winter presents a severe energetic challenge to endothermic animals. Just as food availability drops to its annual minimum, low ambient temperatures increase thermoregulatory costs and food requirements. Many mammals escape this energetic bottleneck by accumulating energy reserves before winter, either in the form of body fat or as a food reserve, and by subsequently expressing bouts of torpor during which they depress body temperature well below their active ‘normal thermic’ level [78,79].

Inter-seasonal variation was observed in the microbial metabolism. We found that Chipmunk could adapt to different dietary carbohydrates from spring to autumn because of the ability of the intestinal microbes (IM) to alter their physiology rapidly. In our results, we observed the relationship of high carbohydrate metabolism with a high abundance of Bacteroides in spring, which decreases in autumn. Our findings have consistency with the previous studies that showed an increased number of saccharolytic bacteria, including Bacteroides and bifidobacteria, was associated with high carbohydrate diets, which have been individually connected with improved regulation of body energy [80]. We have shown that chipmunks have a much higher carbohydrate metabolism in spring than in summer and autumn, but carbohydrate metabolism is similar between summer and autumn [81]. 

We observed that *Bacteroides* were high in abundance in summer, which might involve protein metabolism, because we also observed more amino acid metabolism in summer than in other seasons. Our results were supported by a previous report obtained from human studies, which revealed that *Bacteroides* tend to be richer in those eating diets high in protein and animal fats [82]. Our results advocate the relationship of Prevotella with carbohydrate metabolism, as we found both in a higher ratio in spring than in other seasons. Another study also supported that a high abundance of Prevotella was reported in those eating high carbohydrate diets [82].

It is documented that animals that consume meat have increased protein, a fat component, and a low level of bre content, which is the opposite of animals that depend on a vegetable-based diet by having increased Bre and lessened protein and fat contents. Correspondingly, Faecal acetate and butyrate content were examined more in animals that depend on plants and a difference was observed in metabolite production where an animal-based diet led to an increase in isovalerate and isobutyrate. The level of faecal deoxycholic acid (DCA) was significantly increased due to the animal-based diet, a secondary metabolite product of bile acids resulting from microbial metabolism. The increased concentrations of DCA may have boosted the disturbance of microbial abundance in the animal-based diet, as the growth of Bacteroidetes and Firmicutes phyla members can be inhibited by bile acid production in the host. The animal-based diet increased the abundance of Alistipes, Bilophila, and Bacteroides, which is consistent with the higher bile acid secretion induced by, the higher fat intake [83]. Further, It reduced the levels of Firmicutes such as Roseburia, E.irectale, and R.ibromii that metabolize dietary plant polysaccharides. Previously, variation in intestinal microbiota due to diet shifting from an animal to a vegetable-based diet was investigated through 16S rRNA Illumina sequencing. The results revealed the significant increase in microbiome diversity associated with a vegetable-based diet and were unique to the animal-based diet. Correspondingly, results were reversed 2 days after the end of the diet shifting trail. The animal-based diet was also related to increased expression of bacterial genes for vitamin biosynthesis, degradation of polycyclic aromatic hydrocarbons, and increased expression of ß-lactamase genes [84]. Our study supports the previous findings as PICRUSt results over a three-season period revealed that an omnivorous chipmunk’s metabolism is positively associated with gut microbiota composition and dietary component availability.

## 5. Conclusions

Based on the preliminary findings obtained in this study, we can conclude that the seasonal shift in the Siberian chipmunk diet leads towards an alteration in gut microbiome diversity and host metabolism profile. We believed that our study would help us understand how chipmunk feeding patterns affect community composition. We believe that knowledge of a species’ dietary preferences is essential for understanding its natural history. Furthermore, the current urge to look at animal food is more common during environmental inspections for massive habitat alterations, such as constructing roads and harvesting commercial timber. To lessen the consequences of these activities, biologists must first identify the critical food and cover supplies in an impacted region. Moreover, food analysis is crucial to determining an organism’s fitness, especially for survival and successful reproduction. In addition, our findings will help with understanding how the entire ecosystem interacts and will help conservationists in their efforts to manage and protect the animals. Another finding from our study on gut microbial profiling will help to understand that a chipmunk is a successful individual because the seasonal variation of gut microbiomes advocates the expanded ecological capabilities of the chipmunk, which shows maximum fitness advantage in the changing environment. Previous studies and our findings advocate that diet causes changes in the intestinal flora. In our research, PICRUSt further supports our study by exposing that there were seasonal changes in the assimilation of nutrition. We found that over a three-season period, an omnivorous chipmunk’s metabolism is positively associated with gut microbiota composition and dietary component availability.

## Figures and Tables

**Table 1:** The standard latitudes, longitudes, and altitudes of the study area.

Study Area	Latitude	Longitude	Altitude
Site 1 (iron tower)	47°10′59.58″	128°53′50.89″	405 m
Site 2 (slope 2)	47°10′55.12″	128°53′40.31″	416 m
Site 3 (slope 1)	47°10′54.54″	128°53′40.74″	374 m
Site 4 (footpath)	47°10′57.92″	128°53′42.02″	419 m
Site 5 (log cabin)	47°10′58.93″	128°53′47.21″	452 m

**Table 2:** Sequences of the PCR primer pairs were used to determine the prey of chipmunks in this study. The length of amplified fragments (excluding primers) was between 250 bp to 500 bp.

DNA Marker	Target Group	Primer Name	Primer Sequences (5–3′)	Amplifying Base Pair	References
rbcL	Universal plant mini-barcode	Z1aF/hp2R	ATGTCACCACCAACAGAGACTAAAGC CGTCCTTTGTAACGATCAAG	250 bp	[49]
COI gene	Universal animal mini-barcode	mlCOIintF/REV	GGWACWGGWTGAACWGTWTAYCCYCC TANACYTCNGGRTGNCCRAARAAYCA	360 bp	[50]
ITS Primer Pairs	Fungi	ITS5F/ITS2R	GGAAGTAAAAGTCGTAACAAGG GCTGCGTTCTTCATCGATGC	280 bp	[51]
16S rRNA	microbiota	338F/806R	ACTCCTACGGGAGGCAGCA GGACTACHVGGGTWTCTAAT	500 bp	[52]

## Data Availability

Not applicable.

## Supplementary Materials

The following supporting information can be downloaded at: https://www.mdpi.com/article/10.3390/ani12192586/s1, File S1: Changes in the composition of diet at Phylum and Genus level over seasons, as indicated by genes sequencing data, File S2: Changes in the composition of the Gut microbiome at Phylum and Genus level over seasons, as indicated by genes sequencing data, File S3: Similarity percentages (SIMPER) results exposed the relationship between the seasonal consumption of prey items in chipmunk’s diet, File S4: Similarity percentages (SIMPER) and ANOSIM Results exposed the relationship between the seasonal metabolic capacities.
