# Supplementary figures and images for: Correlation and Influence of Seasonal Variation of Diet with Gut Microbiota Diversity and Metabolism Profile of Chipmunk
Source: Animals (Basel). 2022 Sep 27;12(19):2586. doi: 10.3390/ani12192586 (PMC9559678; doi:10.3390/ani12192586)

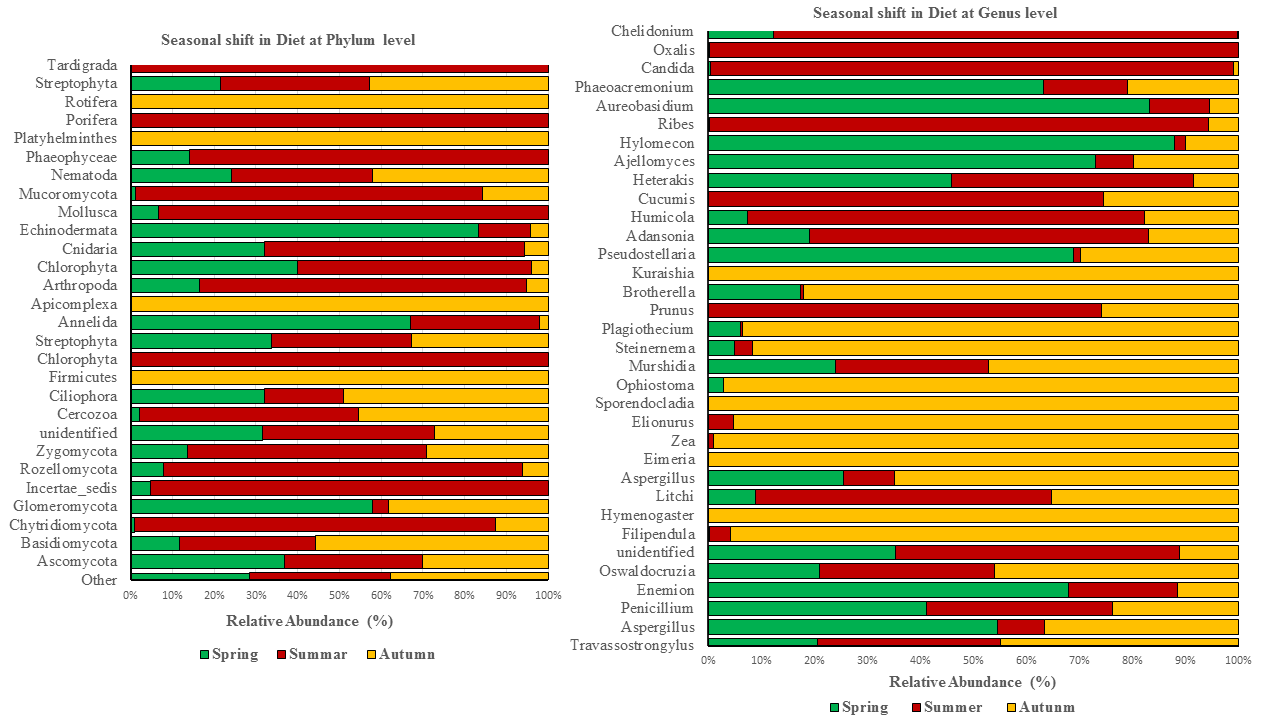

Supplement: Supplementary file 1 [file animals-12-02586-s001.zip › Additional file/File S1.tif]

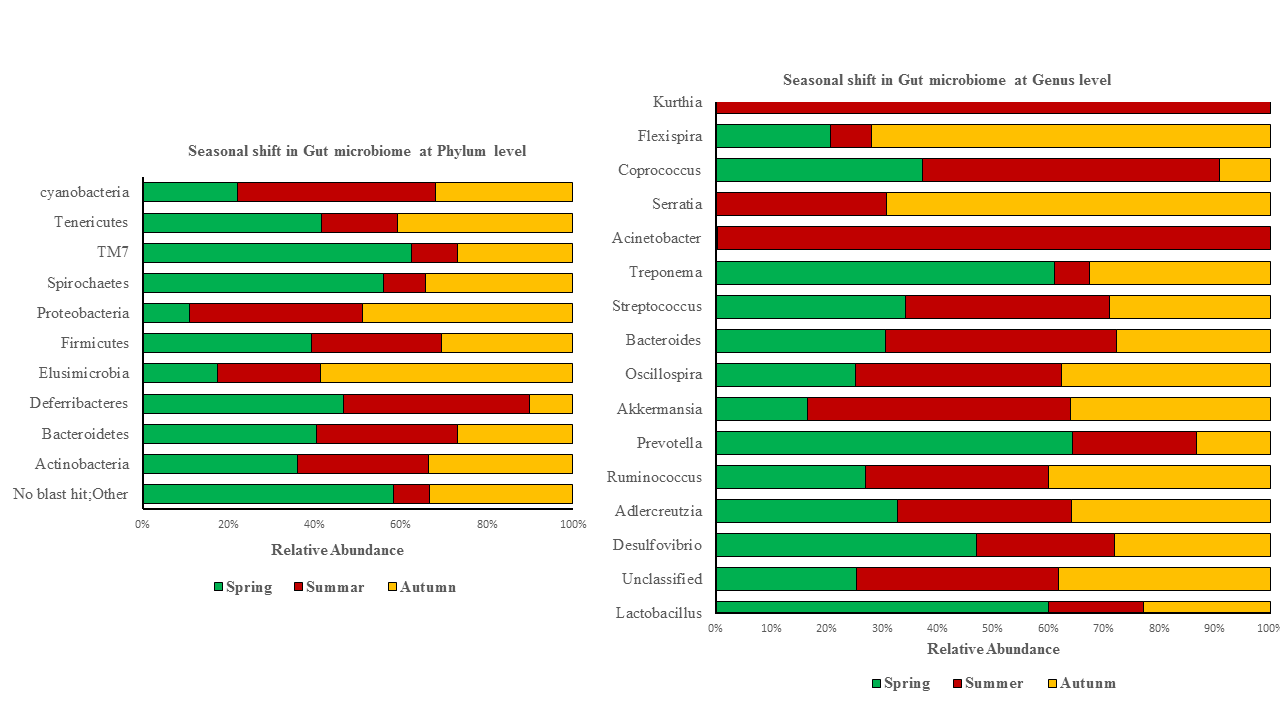

Supplement: Supplementary file 1 [file animals-12-02586-s001.zip › Additional file/File S2.tif]
